# A 36 kg Giant Ovarian Fibroma with Meigs Syndrome: A Case Report and Literature Review of Extremely Giant Ovarian Tumor

**DOI:** 10.1155/2021/1076855

**Published:** 2021-08-15

**Authors:** Miyu Tanaka, Koji Yamanoi, Sachiko Kitamura, Naoki Horikawa, Yoshitsugu Chigusa, Akihito Horie, Ken Yamaguchi, Junzo Hamanishi, Eiji Kondoh, Masaki Mandai

**Affiliations:** Department of Gynecology and Obstetrics, Graduate School of Medicine, Kyoto University, 54 Shogoinkawahara-cho, Sakyo-ku, Kyoto city, Kyoto, Japan 606-8507

## Abstract

Ovarian tumors can get extremely giant to occupy the whole abdominal cavity. We report a case of 36 kg solid ovarian tumor, which was the largest ovarian solid tumor that have been ever reported. A 54-year-old woman presented to our hospital with a chief complaint of markedly distended abdominal wall. Preoperative imaging examinations revealed that most of the tumor was uniform and its density was like that of subcutaneous fat. Pleural effusion was detected in the right thoracic region. We organized a multidisciplinary team and successfully resected the right adnexa. The patient had an uneventful postoperative course, and she was discharged on the 7^th^ postoperative day and diagnosed with a fibroma of the ovary with Meigs syndrome. A comprehensive literature search revealed 48 cases of extremely giant ovarian tumor in these 20 years. Six out of 48 cases are solid. Twelve out of 48 cases are malignant or borderline malignant, and patients' age and tumor size/weight were not related to the frequency of malignancy/borderline malignancy. As many as 4 out of 48 patients died before their first hospital visit or early after surgery. Clinicians should consider a considerable high mortality and frequency of severe surgical complications when planning the treatment strategy for extremely giant ovarian tumors.

## 1. Introduction

Various kinds of tumors can develop in the ovaries, and they can become extremely large, occupying the whole abdominal cavity. However, since extremely giant ovarian tumors (ExG-OvTs) are rare, most of relevant reports are just case reports and there are few reports that investigated a certain number of ExG-OvTs [1]. As for solid ExG-OvTs, there have been only a few case reports [2–6]. ExG-OvT patients experience many symptoms, including a marked decrease in activities of daily living, malnutrition, dehydration, and dyspnea [2, 7–17]. The definitive treatment for ExG-OvTs is surgery, and it is highly assumed that a detailed preoperative assessment should be required. However, the time available for preoperative examinations and the examination methods are very limited because of their strong physical complaints. If we know the clinicopathological background, mortality, and the frequency of severe complication during postoperative period of ExG-OvTs in general, it should help us to consider the treatment strategy and to explain to patients.

Herein, we report a case of a patient with a giant ovarian fibroma with pleural effusion due to Meigs syndrome. To our best knowledge, our case is the largest solid ovarian tumor that has ever been reported. In addition, we performed a literature review to investigate the clinicopathological backgrounds, mortality, and the frequency of severe complication during postoperative period of ExG-OvTs in these 20 years. Our review is the first review that investigates a certain number of ExG-OvTs including solid tumors.

## 2. Case Presentation

A 54-year-old woman (gravida 0, para 0) was transferred to our department with an extensively distended abdominal wall and leg pain. Regular menstruation started at age 14, and she experienced menopause at age 48. She had no history of regular hospitalizations. Over the past few years, she had noticed a gradual progression of abdominal bloating, but she had not decided to go to the hospital. Finally, when it became difficult for her to walk by herself, she went to a nearby hospital and was transferred to our department.

**Figure 1:**
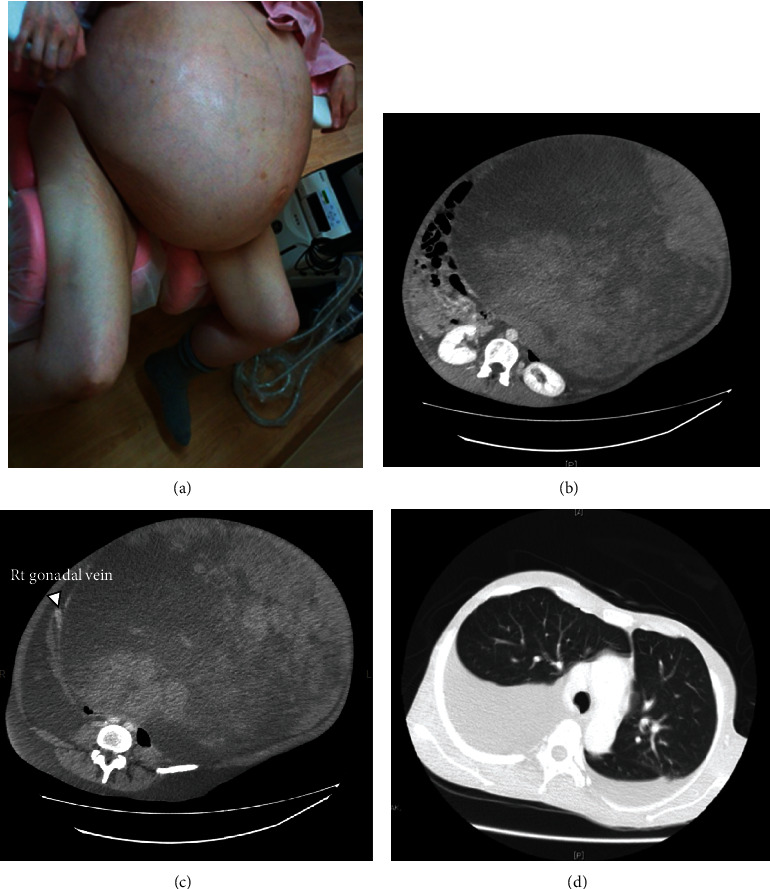
Physical and imaging test findings before operation. (a) Her abdomen was markedly distended, making it difficult for her stand by herself. (b, c, d) Findings of enhanced-computed tomography. (b) Majority of the mass was homogenous with a density of subcutaneous fat and no obvious hypervascular lesions. (c) The right gonadal artery and vein flow into the tumor. (d) The pleural effusion was detected in right thoracic predominance.

Her vital signs were stable; however, her abdomen was markedly distended from the cardiac fossa to the lower abdomen, making it difficult for her to stand by herself (Figure 1(a)). Marked pitting edema was found in both legs.

### 2.1. Imaging Examinations

Contrast-enhanced computed tomography showed that the tumor occupied the whole abdominal cavity (38 cm × 40 cm × 48 cm), and both kidneys were being pressed significantly dorsally (Figure 1(b)). Most of the tumor was uniform, and its density was like that of subcutaneous fat. There were no hypervascular lesions, and the right ovarian artery and vein flowed into the tumor (Figure 1(c)). No obvious venous thrombosis was detected; however, pleural effusion was detected in the right thoracic region (Figure 1(d)). The tumor was too large to obtain useful information from magnetic resonance imaging.

Blood test results showed that the CA125 value was slightly elevated, and there was a marked increase of estradiol and a marked suppression of luteinizing hormone and follicle-stimulating hormone levels (Table 1(a)), which indicated a benign ovarian solid fibroma or thecoma with Meigs syndrome.

We planned to surgically remove the right adnexa, but because of concerns about potentially severe complications, we organized a multidisciplinary team of general surgeons, anesthetists, radiation oncologists, and plastic surgeons to plan the treatment course.

**Figure 2:**
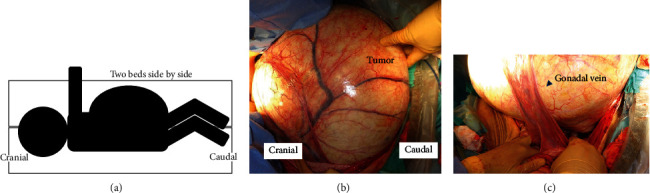
Intraoperative findings in the laparotomy. (a) The surgery was performed in the left lateral recumbency position. (b) Most of the tumor was removed from the body without any adhesions between the tumor and the abdominal wall. (c) The right ovarian artery was found to be flowing into the tumor.

We placed two surgical beds side by side for the operation. During the laparotomy, the patient was placed in the left lateral decubitus position to maintain hemodynamic stability and because the tumor was assumed to be of right ovarian origin (Figure 2(a)). With the help of general surgeons, we confirmed that there were no adhesions between the tumor and the abdominal wall, and the surface of the tumor was smooth (Figure 2(b)). We confirmed that the right ovarian artery and vein truly flowed into the tumor (Figure 2(c)). Her uterine and left adnexa were intact. We cut both vessels, the right fallopian tube, and the ovarian intrinsic ligament and successfully removed the right adnexa. The tumor weighed 36 kg. Because the subcutaneous fascia and skin were markedly stretched by the tumor, a plastic surgeon trimmed the excess fascia and skin and reformed the umbilicus. During the operation, the patient's vital signs were fairly stable. The amount of intraoperative blood loss was 420 mL, and the operation time was 4 hours and 17 minutes.

**Figure 3:**
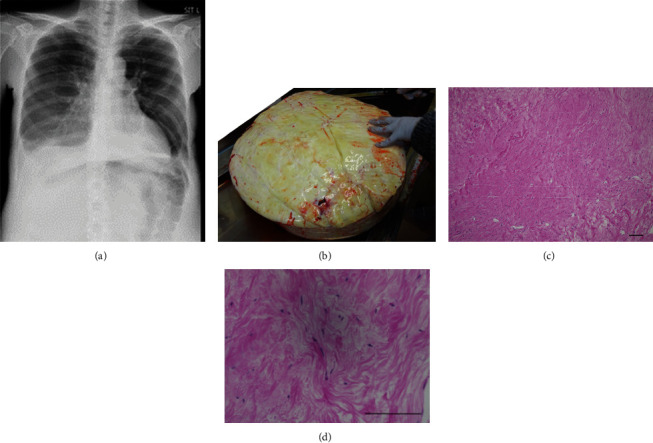
Postoperative findings and pathological findings. (a) Chest X-ray on the fourth postoperative day showed marked decrease of pleural effusion. (b) Macroscopic finding of the tumor. It was nearly white, and there were no obvious necrotic lesions. (c, d) Microscopic findings. The tumor was composed of thin spindle cells in a whorled arrangement, and nuclear atypia and mitosis were not observed. (c) ×40 magnification: black bar is 1 mm. (d) ×100 magnification: black bar is 200 *μ*m.

The patient was then extubated and moved to the intensive care unit for recovery. There were no signs of major complications, and she was moved to the general ward on the 1^st^ postoperative day. A chest radiograph on the fourth postoperative day showed a marked decrease in the right pleural effusion (Figure 3(a)). The postoperative course was generally favorable, and the patient was discharged on the 7^th^ postoperative day.

A pathological examination showed that the tumor was macroscopically nearly white, but there were no obvious necrotic lesions (Figure 3(b)). Microscopically, the tumor was composed of thin spindle cells in a whorled arrangement, but nuclear atypia and mitosis were not observed, and the fibroma diagnosis was confirmed (Figures 3(c) and 3(d)).

On the 29th postoperative day, the patient visited the outpatient, and the wound was observed to be healing well. A blood test performed 7 months after the surgery confirmed that her hormonal status had returned to the menopausal status (Table 1(b)), and she did not show any complaints.

## 3. Discussion

Because there have been no reports of solid ExG-OvTs larger than the present case [2–6], it was initially difficult to understand the patient's pathological condition and determine the proper surgical treatment. Therefore, we conducted a literature search, using the MEDLINE database, of ExG-OvTs to investigate the clinicopathological characteristics and postoperative complications associated with these tumors.

**Figure 4:**
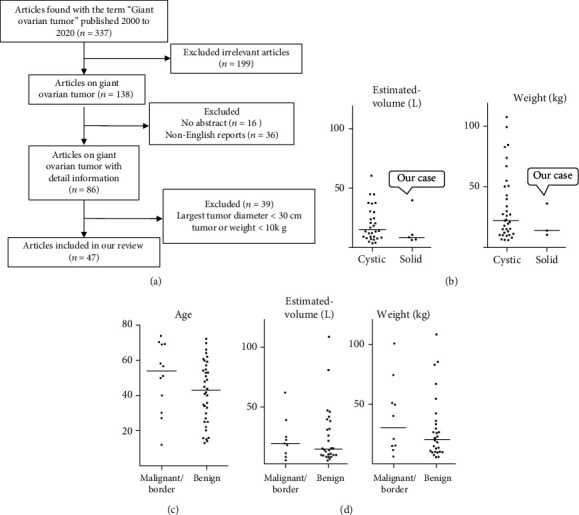
Literature review: flow and comparison of cystic and solid, begin, and borderline/malignancy. (a) Schematic of review. Case reports published from 2000 to 2020 were searched. We defined ExG-OvTs as (1) tumors with a maximum diameter > 30 cm in imaging or (2) tumors weighing more than 20 kg. As a result, 48 cases were reviewed including our case. (b) Comparison of estimated volume (left side) and weight (right side) between cystic and substantial tumors. (c) Comparison of age between benign and malignant/borderline tumors. (d) Comparison of estimated volume (left side) and weight (right side) between benign and malignant/borderline tumors.

We began by searching for case reports published from 2000 to 2020. Our search showed no consensus criteria for extremely giant ovarian tumors. Consequently, we defined ExG-OvTs as (1) tumors with a maximum diameter > 30 cm in imaging or (2) tumors weighing more than 20 kg. These criteria narrowed our results to 47 cases, to which we added our case for all further analyses, for a total of 48 cases (Figure 4(a)). Details about all 48 cases are described in supplementary Table (available here).

**Figure 5:**
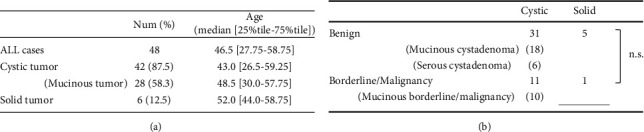
The frequency of cystic/solid tumors and borderline/malignant tumors among extremely giant ovarian tumors (ExG-OvTs). (a) Distribution of cystic/solid ExG-OvTs and their age. (b) The frequency of begin and borderline/malignancy in ExG-OvTs.

The resulting reports showed that ExG-OvTs have been found in patients of a wide age range, from 12 to 74 years. The frequency of solid ExG-OvTs was low (6/48 cases, 12.5%, Figure 5(a)). Eighty-seven percent of the patients had cystic tumors, and mucinous tumors were the most frequent (28/48, 58.3%, Figure 5(a)). Ages and size/weight of tumors did not differ significantly between cystic and solid ExG-OvTs (Figures 5(a) and 4(b)), although size/weight of solid ExG-OvTs are relatively smaller than those of cystic ones. The tumor in the present case was much larger and heavier than those of the other five solid ExG-OvT cases (Figure 4(b), Supplementary Table (available here)).

It was difficult to determine whether the pleural effusion in the present case was due to Meigs syndrome or a malignant tumor. Of the total reports in our analysis, 12/48 cases (25.0%) were of malignant or borderline malignant tumors. There were no significant differences about the frequency of malignant or borderline malignant tumors between the cystic and solid ExG-OvTs (cystic: 11/42, solid: 1/6; Figure 5(b)). There were also no significant differences in the size/weight and patient ages between the benign and malignant/borderline ExG-OvTs (Figures 4(c) and 4(d)).

In our review, we first showed that up to 25% of ExG-OvTs could be malignant or borderline malignant. In addition, their age and tumor size were found not to be related to the frequency of malignancy or borderline malignancy. We should employ several diagnostic approaches, including imaging and physical examinations, to assess the malignancy potential of each case. In the present case, although some tumor markers were moderately elevated, contrast-enhanced computed tomography showed a relatively homogenous area within the tumor and no obvious lymph node enlargement or tumor dissemination lesions. Therefore, a benign tumor with Meigs syndrome was suspected.

Surprisingly, among the reviewed cases, as many as four patients (8.3%) deceased (two died of cardiopulmonary arrest before arrival at the hospital, and two died of fatal postoperative complications (Tables 2(a) and 2(b)) [17–20]. Three of the four patients had pathologically benign tumors (Tables 2(a) and 2(b)). In addition, major surgical complications developed in as many as 6 cases (12.5%), and 4 were pathologically benign and 2 were malignant (Table 2(c)) [2, 21–24]. Therefore, we should take the high preoperative mortality and the high frequency of fatal postoperative complication into account when determining the treatment strategy for ExG-OvTs regardless of their malignant potential. When surgical interventions are planned, it is obviously critical to employ a multidisciplinary team, including anesthesiologists, cardiologists, and general and plastic surgeons [24, 25]. In the present case, the surgery was fortunately completed without severe complications. For cystic tumors, the tumor fluid can gradually be aspirated to prevent rapid hemodynamic changes [11, 26]; however, this is not possible for solid tumors. In the present case, one did not occur. All six solid ExG-OvTs cases were successfully treated surgically, and those can be less prone to hemodynamic instability than cystic ExG-OvTs. Although we placed the patient in the left lateral position to prevent rapid hemodynamic changes, the effect this had is unknown and still to be elucidated.

In conclusion, to the best of our knowledge, we successfully resected the largest solid ovarian tumor that has ever been reported with help of a multidisciplinary team without any severe complications. Although many ExG-OvTs (about 75%) are pathologically benign, they are still strongly associated with a high mortality and fatal postoperative complications, and we have to take that into account when planning the treatment.

## Figures and Tables

**Table 1a:** (a) (a) Results before operation

WBC	6.39 × 10^3^/L	AST	20 U/L
RBC	4.02 × 10^5^/L	GPT	14 U/L
HGB	12.6 g/dL	LDH	266 U/L
PLT	132 × 10^3^/L	Total protein	6.0 g/dL
PT-INR	1.03	Albumin	3.3 g/dL
Fibrinogen	249 mg/dL	Total-Bil	0.6 mg/dL
D-dimer	4.5 *μ*g/mL	Creatinine	0.52 mg/dL
		CK	225 U/L
LH	<0.1 mIU/mL	CRP	0.1 mg/dL
FSH	<0.1 mIU/mL		
Estradiol	43.7 pg/mL	CA125	384.4 U/mL
Progesterone	0.92 ng/mL	CA19-9	5.3 U/mL

**Table 1b:** (b) (b) Results after operation

LH	37.8 mIU/mL
FSH	69.7 mIU/mL
Estradiol	<5.0 pg/mL
Progesterone	0.41 ng/mL

**Table 2a:** (a) (a) Cases of CPR at their first hospital visit

Case no.	Pathology	Age	Estimated tumor volume (L)	Tumor weight (kg)
2	Mucinous cystadenoma	51	8.05	26
9	Mucinous cystadenoma	30	Unknown	67

**Table 2b:** (b) (b) Cases of fatal surgical complications

Case no.	Pathology	Age	Estimated tumor volume (L)	Tumor weight (kg)	Clinical course
14	Mucinous cystadenoma	48	Unknown	Above 30	Death at the 11th postoperative day
34	Mucinous adenocarcinoma	30	Unknown	100	Death 10 hours after operation

**Table 2c:** (c) (c) Cases of major surgical complications

Case no.	Pathology	Age	Estimated tumor volume (L)	Tumor weight (kg)	Context of complications
10	Mucinous cystadenoma	53	45	27	Deep vein thrombosis after operation
15	Mucinous cystadenoma	72	38.25	27	Bowel damage during operation
18	Mucinous cystadenoma	66	Unknown	23	Postoperative ileus, conservative therapy.
32	Mucinous adenocarcinoma	40	Unknown	74	Postoperative infection
33	Mucinous adenocarcinoma	51	Unknown	50.7	Need of reintubation during postoperative period
44	Fibroma	41	8.1	10	Need of bowel resection.

CPR: cardio pulmonary arrest.

## Data Availability

The data that support the findings of this study are available from the corresponding author upon reasonable request. As for literature review, we provide PMID of all reviewed case reports in supplementary table.
