# Supplementary material for: A 36 kg Giant Ovarian Fibroma with Meigs Syndrome: A Case Report and Literature Review of Extremely Giant Ovarian Tumor
Source: Case Rep Obstet Gynecol. 2021 Aug 15;2021:1076855. doi: 10.1155/2021/1076855 (PMC8382553; doi:10.1155/2021/1076855)
Supplement: Supplementary Materials — Supplementary table is available, which includes all clinical information used in the literature review. [file 1076855.f1.pdf]

Supplementary Table: List of reviewed cases.

No.	PMID	Pathology	Benign or not	Cystic/ Solid	age	Diameter (cm)			Tumor-		Complication
						L1	L2	L3	E-size (L)	Weight (kg)	
1	20499763	mucinous cystadenoma	B	C	35					33.6	
2	21659835	mucinous cystadenoma	B	C	51	35	23	20	8.05	26	CPR at the first visit
3	22854108	mucinous cystadenoma	B	C	13	40	30	20	12		
4	25603687	mucinous cystadenoma	B	C	16	45	32	28	20.16	18	
5	25611279	mucinous cystadenoma	B	C	44	59	48	32	45.312	30	
6	26097766	mucinous cystadenoma	B	C	40	30	15	15	3.375	22	
7	26513896	mucinous cystadenoma	B	C	61	47	43	30	30.315	42.5	
8	27134482	mucinous cystadenoma	B	C	42	35	31	13.2	7.161	6	
9	27890095	mucinous cystadenoma	B	C	30					67	CPR at the first visit

10	28913091	mucinous cystadenoma	B	C	53	40	50	45	45	27	Deep vein thrombosis after operation
11	29960208	Mucinous cystadenoma with brenner tumor	B	C	57	40	22	27	11.88		
12	30363636	mucinous cystadenoma	B	C	15	30	30	30	13.5		
13	30956200	mucinous cystadenoma	B	C	53	75	51	42	80.325	108	
14	31196215	mucinous cystadenoma	B	C	48						Death at the 11th postoerative day
15	31672957	mucinous cystadenoma	B	C	72	40	45	42.5	38.25	27	Bowel damage during operation
16	32698286	mucinous cystadenoma	B	C	20	60	60	60	108	10	
17	32913859	mucinous cystadenoma	B	C	49	30	30	30	13.5	10.15	
18	33031322	mucinous cystadenoma	B	C	66					23	Postoperative ileus, conservative therapy.

19	16249904	serous cystadenoma	B	C	36	35	20	26	9.1	9.5
20	18214509	serous cystadenoma	B	C	22	33	26	15	6.435	6
21	18226141	serous cystadenoma	B	C	34					85
22	28840047	serous cystadenoma	B	C	27	31.7	26.3	15.6	6.502938	11
23	31308929	serous cystadenoma	B	C	64	40	34	36	24.48	13
24	31878899	serous cystadenoma	B	C	25	42	42	42	37.044	19.7
25	16882432	Mature cystic teratoma	B	C	16	40.5	30	22.5	13.66875	15
26	18280952	Mature cystic teratoma	B	C	25	32	27	18.5	7.992	10
27	21594602	begign brenner tumor	B	C	62	39	39	39	29.6595	8
28	12971150	benign cyst	B	C	33					55
29	24102044	benign cyst	B	C	59					83
30	30188242	lymphangioma	B	C	60	40	15	29	8.7	
31	19069717	Para-ovarian cyst	B	C	14	30	26	12	4.68	

32	16343196	Mucinous adenocarcinoma	C	C	40					74	Postoperative infection
33	16343263	Mucinous adenocarcinoma	C	C	51					50.7	Need of re-intubation during postoerative period
34	17212674	Mucinous adenocarcinoma	C	C	30					100	Death 10 hours after operation
35	24594205	Mucinous adenocarcinoma	C	C	57	42	40	28	23.52	40	
36	25661539	Mucinous adenocarcinoma	C	C	27	32	35	37	20.72	50	
37	26881612	Mucinous borderline tumor with Brenner tumor	M	C	70	52	40	36	37.44	20.7	
38	30071377	Mucinous borderline tumor	M	C	69	60	50	40	60	6.5	
39	30863733	Mucinous borderline tumor	M	C	74	33	33	33	17.9685	15	
40	31579185	Mucinous borderline tumor	M	C	12	42	22	20	9.24	11.8	
41	33244339	Mucinous borderline tumor	M	C	58	44	39	19	16.302	15.4	

42	26052208	Carcinosarcoma	C	C	69	33	22	10	3.63		
43	19644698	leiomyoma	B	S	55	30	28	15	6.3		
44	31231501	Fibroma	B	S	41	27	30	20	8.1	10	Large amount of ascites,<br>Need of bowel resection.
45	17447642	Fibrothecoma	B	S	45					13.6	
46	18813938	Fibrothecoma	B	S	70	35	30	20	10.5		
47	33011729	Fibrosarcoma	C	S	50	30.2	25.4	16.5	6.32841		
48	our case	fibroma	B	S	54				40	36	

PMID: PubMed ID number.

Benign or not; Benign: B, Borderline malignancy: B. Carcinoma or malignancy: C

Cystic or Solid; Cystic: C, Solid: S

Estimated size: E-size.

Tumor volume is estimated by modified ABC/2 method.

CPR: Cardio-Pulmonary Arrest

Caption: List of review cases including our case
